# Hyperechoic breast images: all that glitters is not gold!

**DOI:** 10.1007/s13244-017-0590-1

**Published:** 2018-02-23

**Authors:** Gabrielle Journo, Guillaume Bataillon, Raphael Benchimol, Asma Bekhouche, Chloe Dratwa, Delphine Sebbag-Sfez, Anne Tardivon, Fabienne Thibault, Catherine Ala-Eddine, Pascal Chérel, Caroline Malhaire

**Affiliations:** 1 Institut Curie, 26 rue d’Ulm, 75005 Paris, France; 2 Centre René Huguenin, 35 rue Dailly, 92210 Saint-Cloud, France

**Keywords:** Breast cancer, Breast ultrasound, Histology, Hyperechogenicity, Malignancy

## Abstract

**Abstract:**

Hyperechogenicity is a sign classically reported to be in favour of a benign lesion and can be observed in many types of benign breast lesions such as hamartoma, lipoma, angiolipoma, haemangioma, haematoma, fat necrosis, fibrosis and galactocele, among others. However, some rare malignant breast lesions can also present a hyperechoic appearance. Most of these hyperechoic malignant lesions present other characteristics that are more typically suggestive of malignancy such as posterior shadowing, a more vertical axis or irregular margins that help to guide the diagnosis. Post magnetic resonance imaging, second-look ultrasound may visualise hyperechoic malignant lesions that would not have been identified at first sight and radiologists must know how to recognise these lesions.

**Teaching Points:**

*• Some rare malignant breast lesions can present a hyperechoic appearance.*

*• Malignant lesions present other characteristics that are suggestive of malignancy.*

*• An echogenic mass with fat density on mammography does not require biopsy.*

## Introduction

The majority of breast lesions detected by ultrasound are hypoechoic. According to the BI-RADS lexicon [1], a hyperechoic lesion is defined by an echogenicity greater than that of subcutaneous fat or equal to that of fibroglandular parenchyma. Only 1–6% of breast masses are hyperechoic and the great majority of them are benign. However, malignant lesions can rarely present in the form of hyperechoic images [2]. Hyperechogenicity has a variable histological origin [3] and has been attributed to the presence of:

- Densely grouped adipocytes
- Thick bands of fibrosis
- Multiple vascular spaces
- A heterogeneous and invasive tumour cell contingent

In their original study published in 1995, based on a series of 750 breast nodules detected by ultrasound, Stavros et al. [4] reported that 42 nodules were hyperechoic and all of them were benign. Hyperechogenicity was the ultrasound parameter in favour of a benign lesion with the highest negative predictive value (100%). In a more recent study published by Linda et al. [3], retrospective review of a series of 4511 biopsied lesions revealed that 25 (0.6%) were hyperechoic and 9 (0.4%) were malignant.

The differential diagnosis of hyperechoic breast images is based on knowledge of the clinical setting, detailed analysis of morphological features, and comparison with mammography and possibly magnetic resonance imaging (MRI). In this paper, we propose a review of the various hyperechoic breast lesions.

## Technical parameters

The American College of Radiology [5] recommends the use of large bandpass linear transducers with a central frequency of at least 10 MHz for breast ultrasound. The frequency is adapted to the nature of the breast volume and the site of the lesion. Total gain and gain at various depths must be modulated to obtain a homogeneous intermediate signal for fat of the premammary zone, mammary zone and retromammary zone. The focal length is adjusted to the depth over the lesion. Two complementary modes can be used:

- Harmonic mode: reduces artefacts, improves spatial resolution and the contrast between glandular tissue, fat and breast lesions by increasing the echogenicity of fat, and enhances posterior ultrasound modifications.
- Compound mode or real-time spatial compound imaging: improves the signal-to-noise ratio and optimises analysis of lesion margins and the internal echostructure of breast masses. Posterior ultrasound modifications are attenuated.

## Benign hyperechoic lesions

### Lipoma

Lipoma is a proliferation of mature adipocytes forming a lobular mass clearly circumscribed by a fine fibrous capsule. Lipoma is a common lesion, often unilateral and solitary, and can present as a soft, mobile palpable mass.

**Fig. 1:**
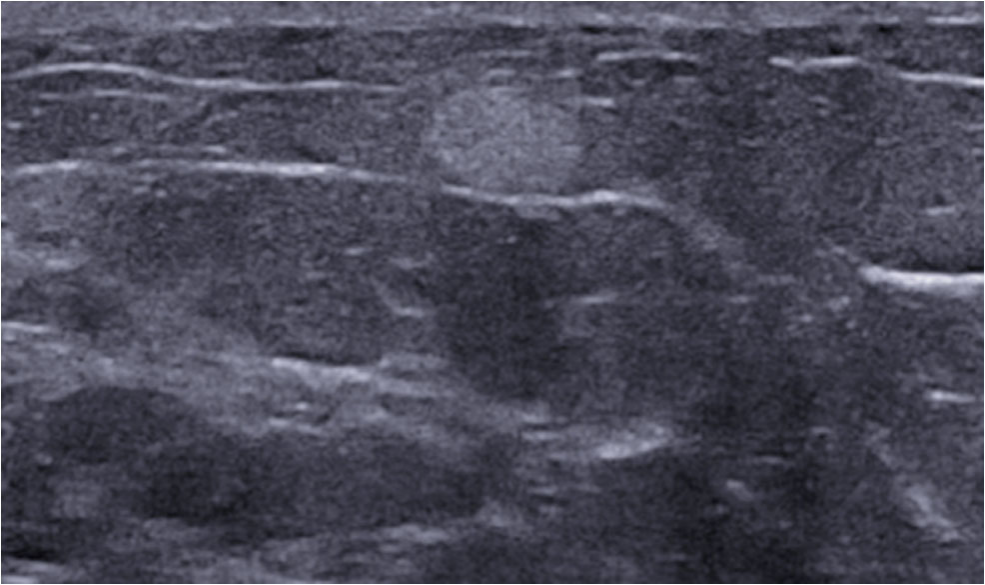
Spindle cell lipoma. Transverse ultrasound scan showing a homogeneous, hyperechoic, round mass with well circumscribed margins

On mammography, lipoma presents as a radiolucent lesion with regular margins, surrounded by a fine radiopaque capsule. On ultrasound, lipoma is a homogeneous lesion with variable echogenicity: isoechoic, similar to subcutaneous fat, or more rarely hyperechoic, due to densely assembled adipocytes (Fig. 1). An echogenic mass with fat density on mammography is benign and does not require biopsy [6, 7].

### Angiolipoma

**Fig. 2:**
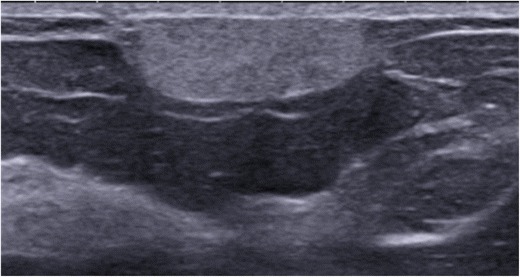
Angiolipoma. Transverse ultrasound scan of the right breast showing a homogeneous, hyperechoic, oval-shaped subcutaneous mass with circumscribed margins

Angiolipoma is a rare benign lesion composed of mature adipocytes associated with a network of small, narrow-lumen vessels typically containing fibrin thrombus. Angiolipoma classically has a superficial topography, in the subcutaneous tissue, and presents as a painless mass [8]. On mammography, angiolipoma presents as a well-circumscribed solid mass or mixed asymmetric density (solid and fat). On ultrasound, angiolipoma is a clearly demarcated, homogeneous, isoechoic to hyperechoic mass with regular margins (Fig. 2). Its superficial topography may be suggestive of the diagnosis, but biopsy is often performed due to its non-specific presentation.

### Fat necrosis

**Fig. 3:**
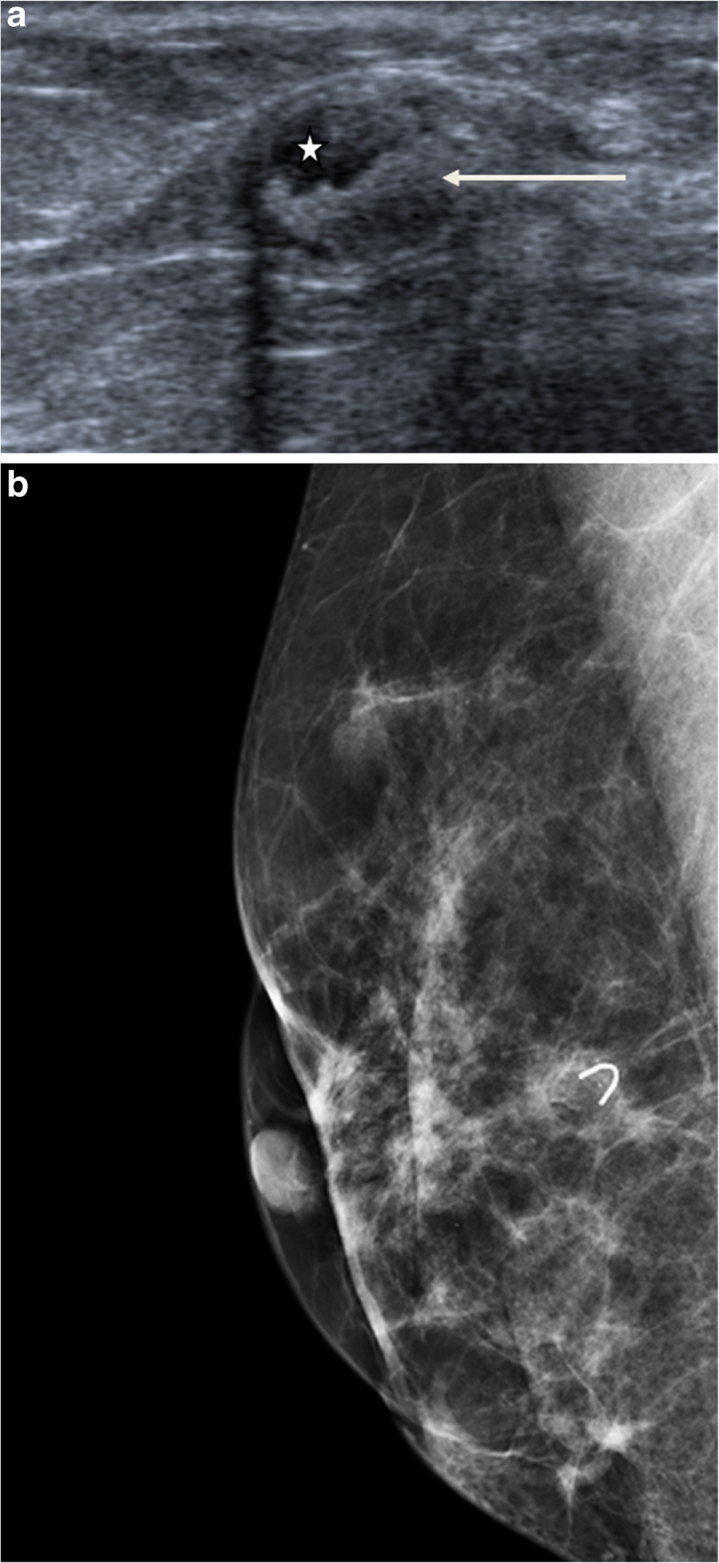
Fat necrosis. **a** Ultrasound scan showing a complex mass composed of an echo-free fat component (*asterisk*) and an echogenic fibrous component (*arrow*). **b** Mammography, oblique view, showing a radiolucent formation projected over a clip

Fat necrosis is the result of direct trauma, infection, surgery or may be secondary to radiotherapy. It can have a variable appearance, comprising areas of macrophage foam cells, siderophages, fibrous changes or even calcification. On mammography, fat necrosis may present as a radiolucent cyst, possibly associated with thick calcifications, a mass or distortion with spiculated or irregular margins. The ultrasound appearance is variable, sometimes hyperechoic, simple cyst, or a complex mass comprising solid and cystic components (Fig. 3a) [9]. The diagnosis can be established by the suggestive clinical setting and demonstration of the fat density content on mammography (Fig. 3b).

### Myofibroblastoma

**Fig. 4:**
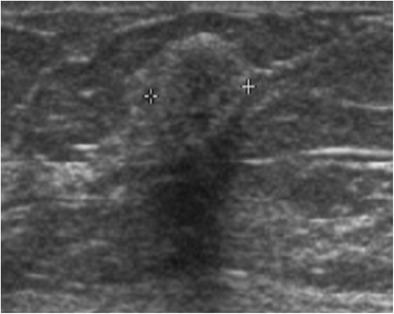
Myofibroblastoma. Ultrasound scan showing a slightly heterogeneous, hyperechoic and attenuating rounded mass

Myofibroblastoma is a rare benign tumour composed of bands of spindle cells, separated by bundles of hyalinised collagen of variable thickness. Myofibroblastoma can be observed in women of all ages, but more commonly in older women. Mammography reveals a well-circumscribed, oval-shaped mass [10], which is rarely calcified. The margins can sometimes appear poorly defined. On ultrasound, it is a solid mass with circumscribed margins that may be either hypoechoic, isoechoic or hyperechoic, suggestive of fibroadenoma. Hyperechoic forms are sometimes attenuating due to their fat content (Fig. 4) [11]. The diagnosis is based on biopsy.

### Haemangioma

**Fig. 5:**
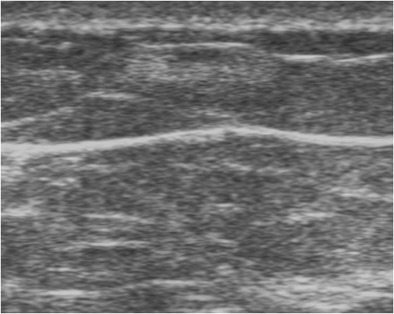
Haemangioma in a 72-year-old woman, presenting with a slowly growing, superficial breast opacity. Ultrasound: oval-shaped, hyperechoic mass of the subcutaneous fat, parallel to the skin. Surgical resection in order to eliminate angiosarcoma: confirmation of the benign nature of the tumour

Haemangioma is a vascular tumour, rarely occurring in the breast, usually observed in middle-aged women. It arises from the breast parenchyma or subcutaneous tissue. On mammography, haemangioma presents as an isodense macrolobular or microlobular lesion with circumscribed margins, possibly containing calcifications. On ultrasound, it is an oval-shaped lesion with circumscribed margins, parallel to the skin with variable echogenicity (hyperechoic in 45% of cases) (Fig. 5). On colour Doppler, haemangioma may be hypovascular with a single feeding artery or hypervascular with multiple feeding arteries [12].

### Haematoma

**Fig. 6:**
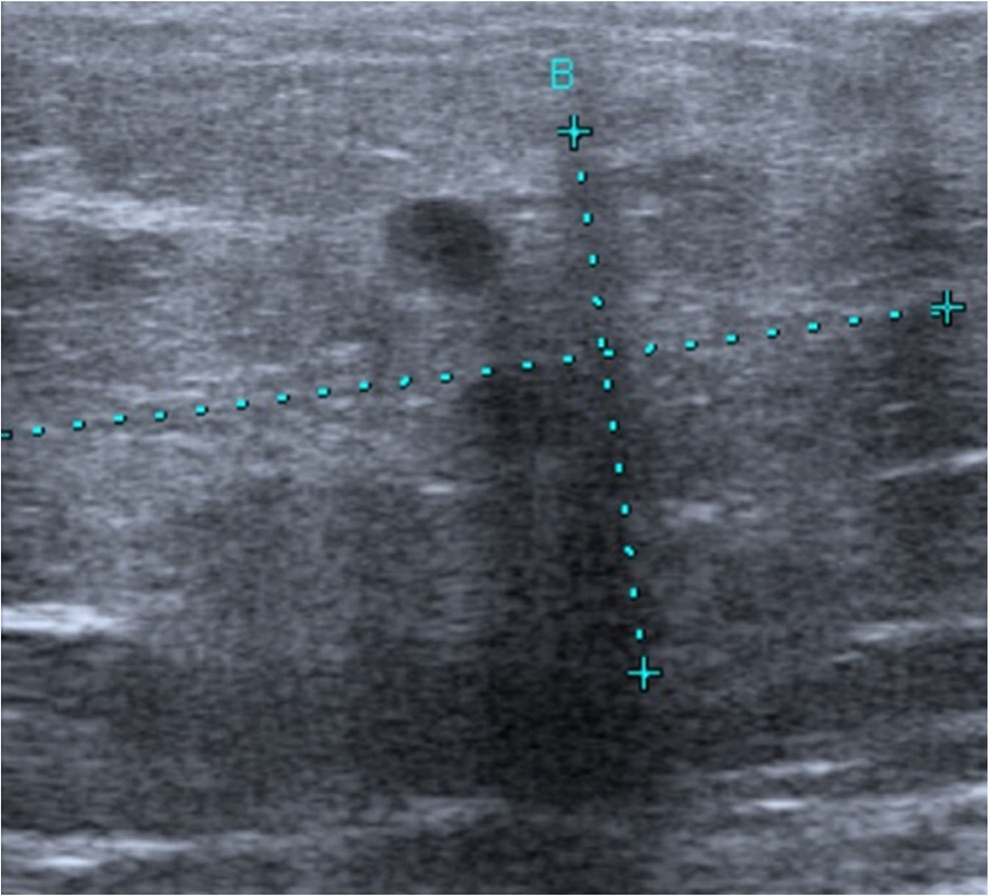
Haematoma. Patient followed for right breast cancer treated in 1998, referred for opinion concerning a mammographic opacity of the right breast not present on the previous mammogram. Ultrasound revealed a mixed hyperechoic and hypoechoic, partially attenuating zone. On clinical interview, the patient reported a motor vehicle accident with ecchymosis of the breast in contact with the safety belt. Ultrasound-guided microbiopsy demonstrated fat necrosis and no suspicious image. Regression of the image on regular follow-up examinations

Haematoma is a localised haemorrhage either secondary to trauma or possibly iatrogenic (interventional procedure, surgery). The ultrasound appearance of haematoma depends on the age of the bleeding: echo-free immediately after the injury, hypoechoic at the acute stage, mixed complex appearance at the subacute stage, hyperechoic at the chronic stage (Fig. 6).

Just-developed hematomas can also be ill-defined and hyperechoic.

The mammographic appearance can be misleading and may mimic malignancy. Correlation with the clinical context and clinical interview are therefore essential for diagnosis.

### Hamartoma

**Fig. 7:**
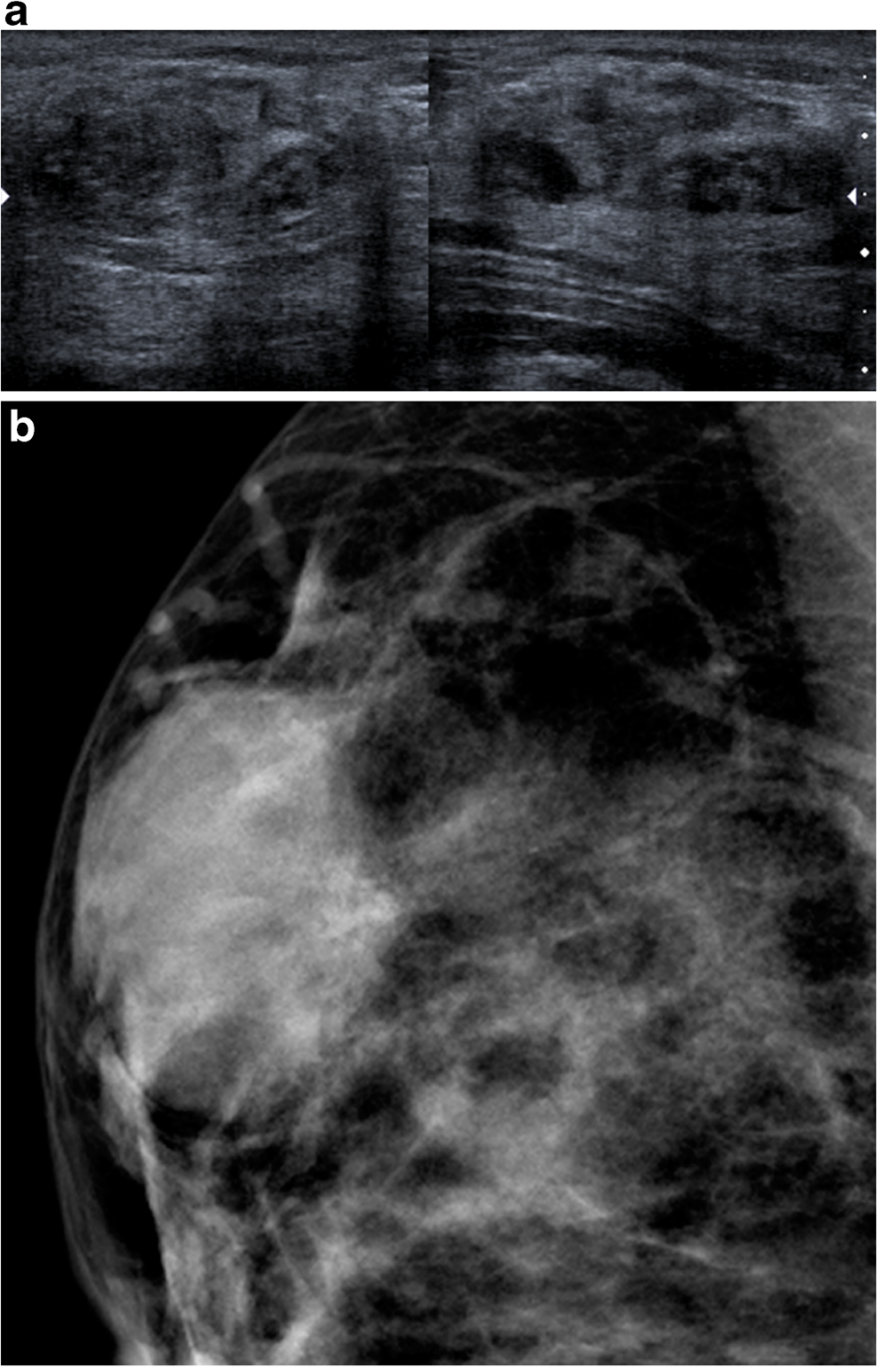
Hamartoma. **a** Ultrasound showing an oval-shaped mass with mixed echogenicity, globally isoechoic, with an edge effect artefact. **b** Mammography showing a well-circumscribed, dense, oval-shaped mass, with a slightly heterogeneous appearance related to its mixed content (fat and stroma)

Hamartoma is a painless, mobile mass, composed of variable proportions of glandular, adipose and connective tissue. Mammography visualises a well-circumscribed, oval-shaped mass containing radiolucent fatty zones, alternating with denser zones corresponding to fibrous tissue, surrounded by a clear pseudocapsule. Ultrasound shows a well circumscribed, oval-shaped compressible mass, surrounded by a fine halo. Hamartomas tend to be hypoechoic, isoechoic or mixed, but can sometimes have a hyperechoic appearance depending on the proportion of the various components. Around 12–43% of hamartomas may appear hyperechoic [6, 13]. An acoustic shadow, a mixture of acoustic shadow and attenuation, or an edge effect artefact may be observed (Fig. 7a and b).

### Diseases of lactating women

#### Galactocele

Galactocele corresponds to milk retention in a dilated lactiferous duct proximal to duct obstruction, generally occurring during breastfeeding or shortly after stopping breastfeeding.

Galactocele can also occur in the absence of breastfeeding in women treated by neuroleptic drugs. The appearance of galactocele depends on the fat and protein content.

**Fig. 8:**
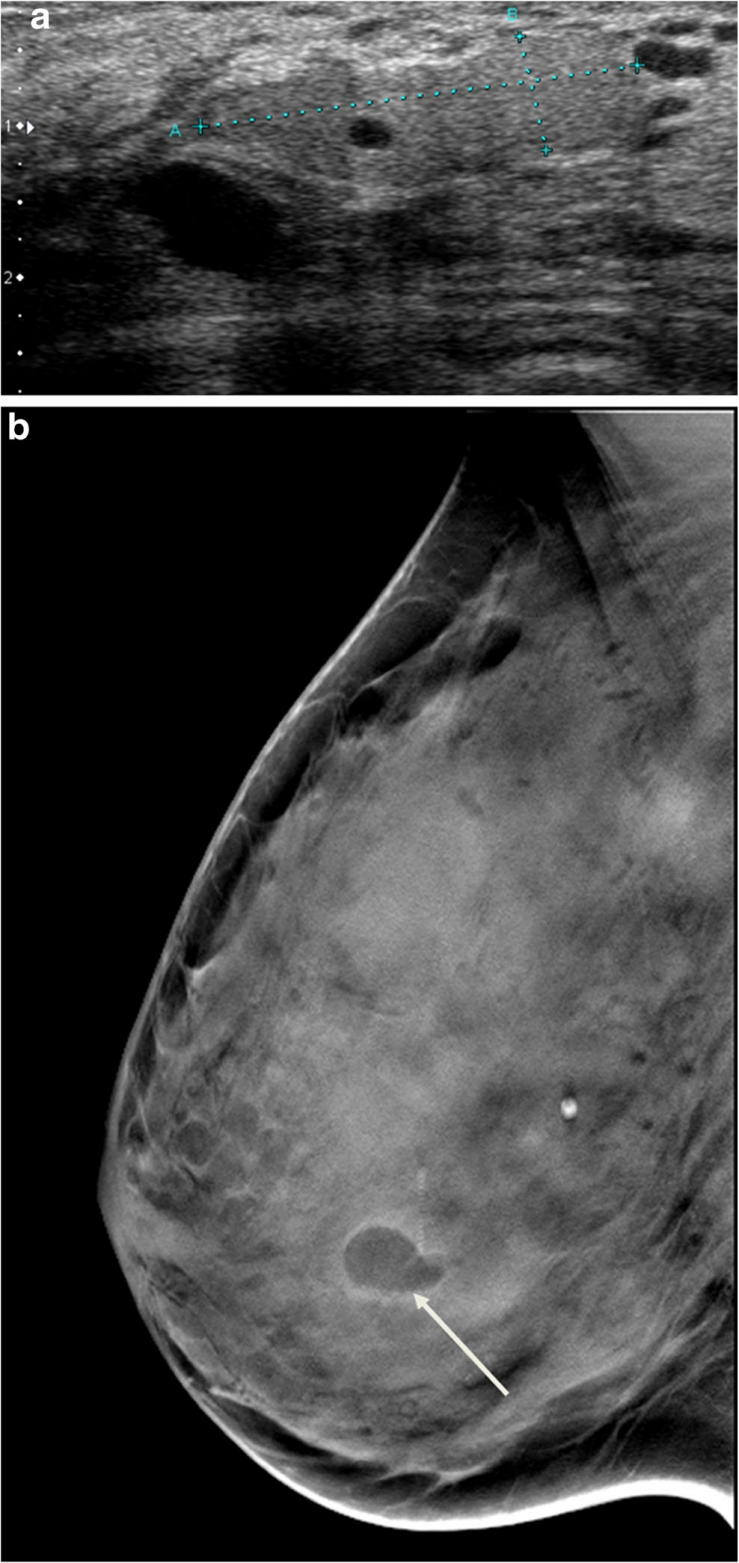
Galactocele. **a** Ultrasound scan showing duct dilatation with homogeneous hyperechoic content, with no blood supply demonstrated by colour Doppler, in a schizophrenic patient with long-term neuroleptic treatment. **b** Digital breast tomosynthesis in the same patient showing a fat density galactocele (*arrow*)

A galactocele with fat density on mammography has a hyperechoic appearance on ultrasound (Fig. 8a and b).

#### Lactating adenoma

Lactating adenoma is a benign tumour occurring during the last trimester of pregnancy, composed of dilated tubular structures, forming alveoli of variable size, lined by vacuolated cells containing lipid-rich foamy material in their centres. Mammography shows an oval-shaped mass containing zones of fat, while ultrasound reveals a well-circumscribed, homogeneous, hypoechoic (67%), isoechoic (20%) or hyperechoic (13%) oval-shaped mass. Lactating adenoma usually resolves spontaneously after stopping breastfeeding [14].

### Pseudoangiomatous stromal hyperplasia (PASH)

Pseudoangiomatous stromal hyperplasia is a benign mesenchymal lesion, more commonly observed in premenopausal women or in women treated by hormonal therapy. PASH consists of proliferation of myofibroblasts induced by a high density of progesterone receptors, leading to the development of fibroblastic hyperplasia. PASH is characterised by the presence of a network of anastomotic channels lined by flat, endothelium-like cells, simulating a vascular tumour. These empty channels, devoid of erythrocytes, predominantly present a concentric pattern around lobules and are typically situated in a dense collagen stroma.

**Fig. 9:**
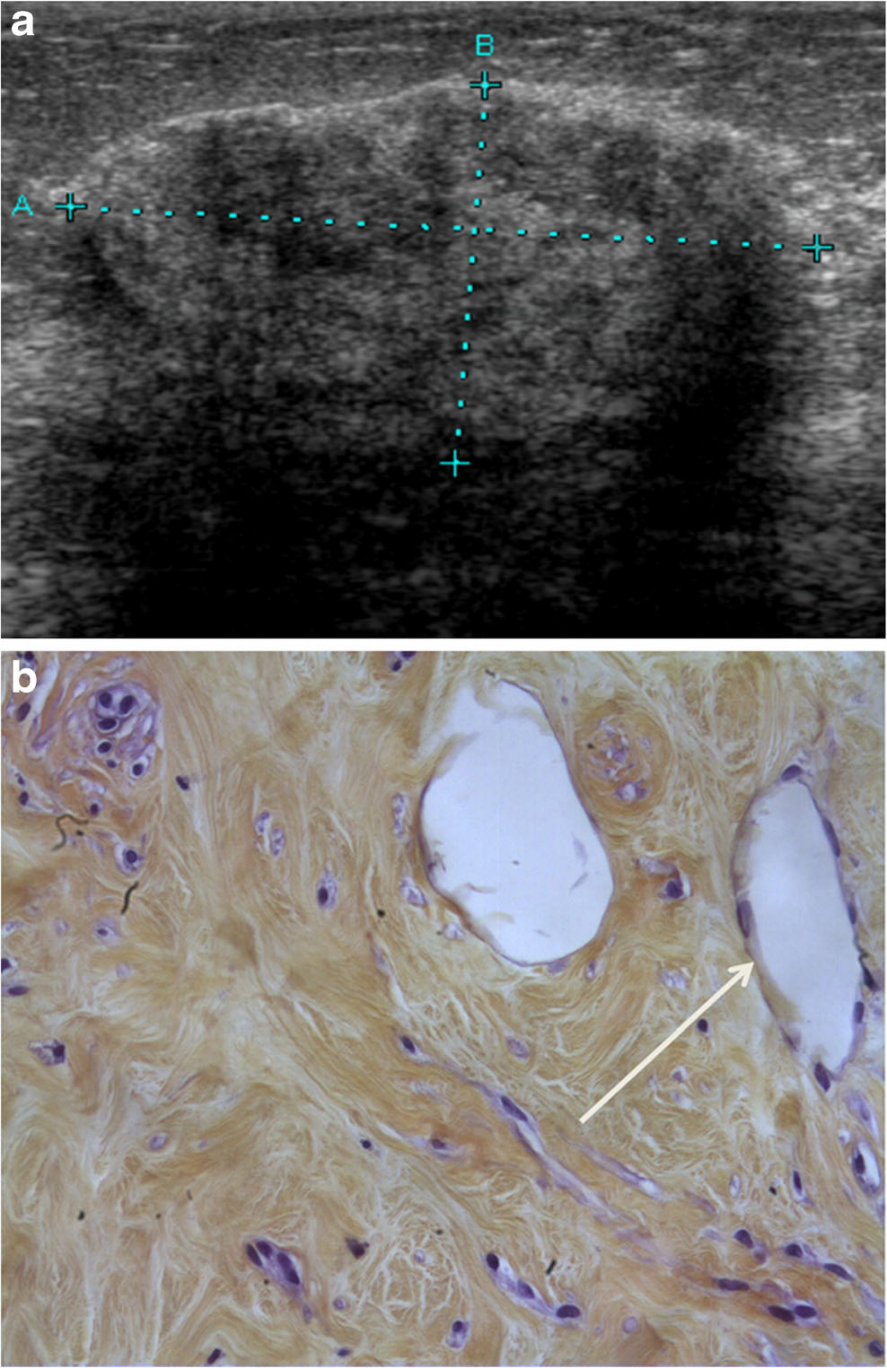
PASH. **a** Ultrasound scan showing a hyperechoic, slightly heterogeneous and attenuating oval-shaped mass. **b** Microscopy (HES ×20): histological section showing fibrosis with pseudoangiomatous stromal hyperplasia (*arrow* showing a channel lined by flat endothelium-like cells)

PASH may present either as a nodular form (palpable or impalpable) or a diffuse form. The nodular form is rare, observed on less than 1% of breast biopsies. Mammography reveals a well-circumscribed, non-calcified mass or asymmetric density. Ultrasound shows a well-circumscribed, hypoechoic and/or hyperechoic oval-shaped mass (Fig. 9b and b).

### Mastitis and breast abscess

**Fig. 10:**
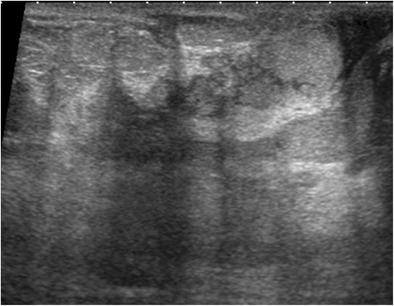
Abscess. A 42-year-old woman presenting with enlargement of the left breast for 1 month with pain and erythema related to mastitis of the left breast. Ultrasound scan of the left breast in the same patient revealing a mixed echogenicity, globally hyperechoic abscess

Breast abscesses are observed in 5–11% [15] of breastfeeding women with infectious mastitis, generally caused by penetration of *Staphylococcus aureus* via a cracked nipple. Breast abscess presents with fever, diffuse or localised erythema of the breast, painful induration and leucocytosis. Smoking increases the risk of breast abscess, which can also occur in non-lactating women (secondary to duct ectasia, cystic inflammation or duct metaplasia) and, more rarely, in men. Mammography shows a well-circumscribed or masked mass, while ultrasound reveals distended ducts with an echogenic content. Although breast abscess may sometimes have an hyperechoic appearance, it generally presents mixed echogenicity (Fig. 10). The diagnosis is usually guided by the clinical context and the favourable course in response to antibiotics. When a breast abscess in a non-lactating woman fails to respond to antibiotics, biopsy should be performed to exclude inflammatory breast cancer (differential diagnosis).

### Siliconoma

**Fig. 11:**
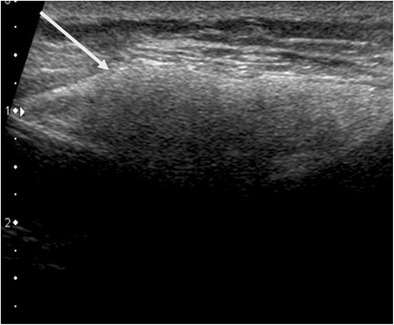
Siliconoma. Periprosthetic silicone (*arrow* showing a hyperechoic mass with attenuation of the ultrasound beam)

Siliconoma is an inflammatory resorption granuloma arising in contact with droplets of free silicone gel, related to capsular rupture or free silicone injections. On mammography, siliconoma presents as a mass isodense to prosthetic silicone. Ultrasound shows a hyperechoic mass containing fine echoes with marked attenuation of the ultrasound beam, with a “snowstorm” appearance masking all posterior structures (Fig. 11). The diagnosis is guided by the clinical context and the ultrasound features, without the need for biopsy.

### Complex sclerosing lesion

**Fig. 12:**
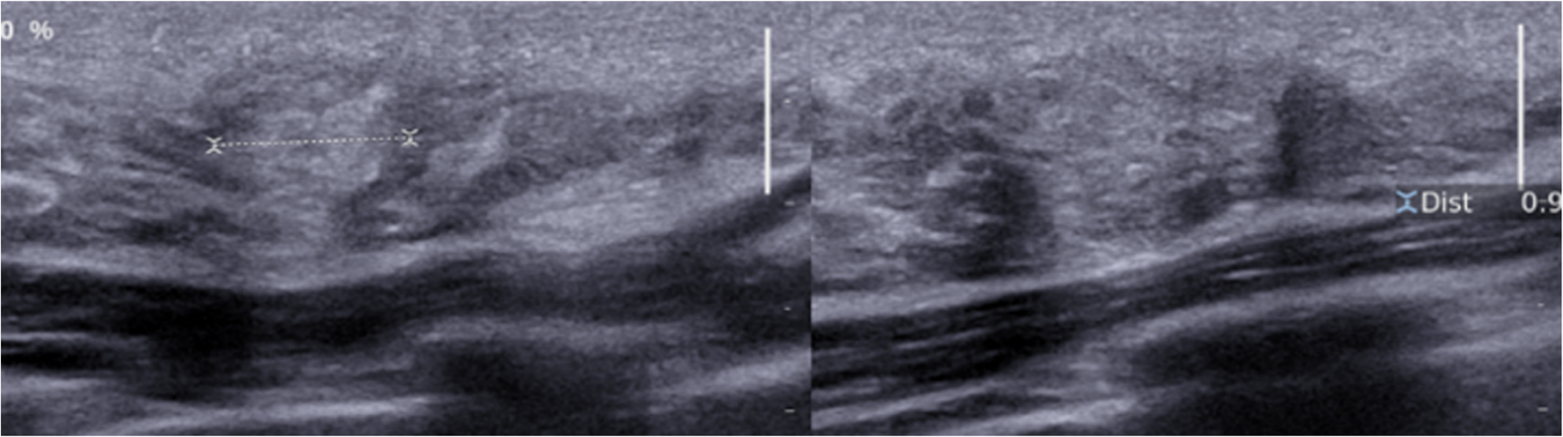
Complex sclerosing lesions. Ultrasound in a young woman with a palpable mass, revealing a hyperechoic zone with irregular margins and acoustic shadow. Microbiopsy demonstrating benign complex sclerosing lesions comprising duct structures and hyperplastic epithelium with no suspicious atypia, together with several pseudopapillary structures, and locally inflammatory periductal fibrosis

Complex sclerosing lesions are benign breast lesions comprising a combination of sclerosing lesions with a variety of proliferative epithelial lesions. Radial scar is a sclerosing lesion with a fibro-elastotic centre surrounded by a radial crown composed of sometimes cystic lobules and ductules, sometimes presenting proliferative lesions. In 30% of cases [16], radial scar is associated with ductal carcinoma in situ or a tubular carcinoma. On ultrasound, it presents as a poorly circumscribed, round or lobular zone, inducing architectural distortion with variable echogenicity, more often hypoechoic, sometimes associated with posterior attenuation (Fig. 12). The diagnosis of these lesions must be based on histological examination of a percutaneous biopsy. When histological examination reveals a diagnosis of associated cancer, the tumour can be treated immediately (including sentinel node biopsy in the case of invasive cancer). When histological examination is negative for cancer, surgical exploration is indicated to avoid missing a focal malignant lesion not detected by percutaneous biopsy.

### Adenosis

**Fig. 13:**
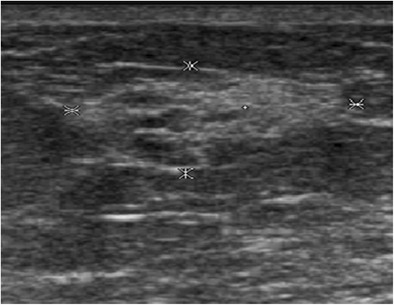
Adenosis. Internal swelling of the right breast with a diameter of 15 mm in a 45-year-old woman, corresponding to a poorly demarcated mammographic opacity associated with punctate microcalcifications, evolving very slowly over a period of 4 years. Ultrasound: zone of mixed echogenicity, predominantly hyperechoic, with a long axis parallel to the skin, not modifying the ultrasound beam

Adenosis corresponds to hyperplasia of all constituents of the terminal duct lobular unit (epithelial cells, myoepithelial cells and connective tissue), resulting in increased size and number of lobules. Adenosis may present clinically in the form of slowly growing breast swelling. In most cases, it is not associated with any mammographic signs. Florid adenosis can present with a multimicronodular radiographic appearance. Punctate microcalcifications may also be observed. On ultrasound, adenosis presents as a zone of variable echogenicity, possibly with irregular margins (Fig. 13).

## Possibly hyperechoic malignant lesions

Breast cancers, regardless of their histology, are typically hypoechoic. Breast cancer can rarely be hyperechoic, but other ultrasound features are usually also present, suggesting the diagnosis, such as poorly circumscribed margins, irregular shape, posterior attenuation, a more vertical axis [17]. Ultrasound characterisation must take the most pejorative parameter into account.

### Invasive ductal carcinoma (IDC)

**Fig. 14:**
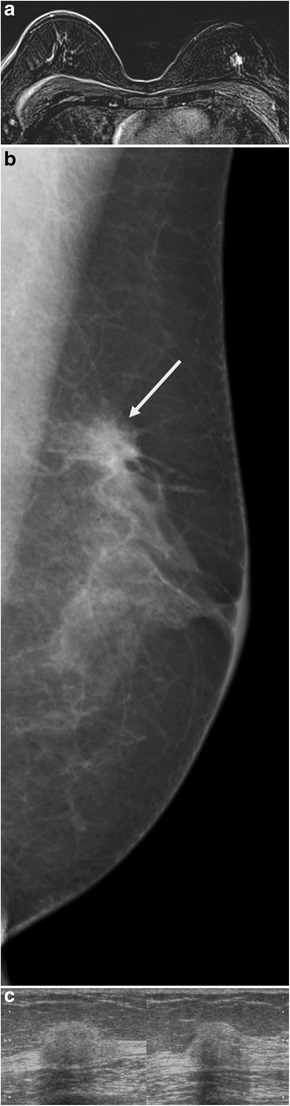
Invasive ductal carcinoma. Annual surveillance in a patient at very high risk already treated for left breast cancer. **a** MRI, gadolinium-enhanced T1 subtraction sequence, demonstrating an irregular, poorly circumscribed mass at the junction of the upper quadrants of the left breast. The gadolinium-enhanced examination shows initial 90% enhancement with washout at the late phase. **b** Mammography guided by MRI in the same patient, oblique view: demonstration of a non-calcified mass (*arrows*), with architectural distortion at the junction of the upper quadrants of the left breast. **c** MRI-guided ultrasound of the left breast in the same patient. At the junction of the upper quadrants, at the 12.30 position, 4 cm from the nipple, presence of a hyperechoic nodule with microlobular margins, with posterior attenuation classified as high ACR4. Microbiopsy demonstrated poorly differentiated invasive ductal carcinoma (grade II, low MI, HR+, HER2-)

IDC accounts for 75% of all breast cancers. On mammography, IDC presents in the form of a spiculated mass, asymmetric density or architectural distortion. On ultrasound, IDC classically presents as a hypoechoic mass, although a hyperechoic or mixed echogenicity lesion may be observed (Fig. 14). In the series published by Skaane et al. [2], 4/194 (2%) of IDCs were hyperechoic. The mechanisms responsible for hyperechogenicity have not been clearly elucidated, but may be related to tumour heterogeneity or the invasion-front-associating thick bands of collagen, adipose tissue and tumour cell proliferation.

### Mucinous carcinoma

**Fig. 15:**
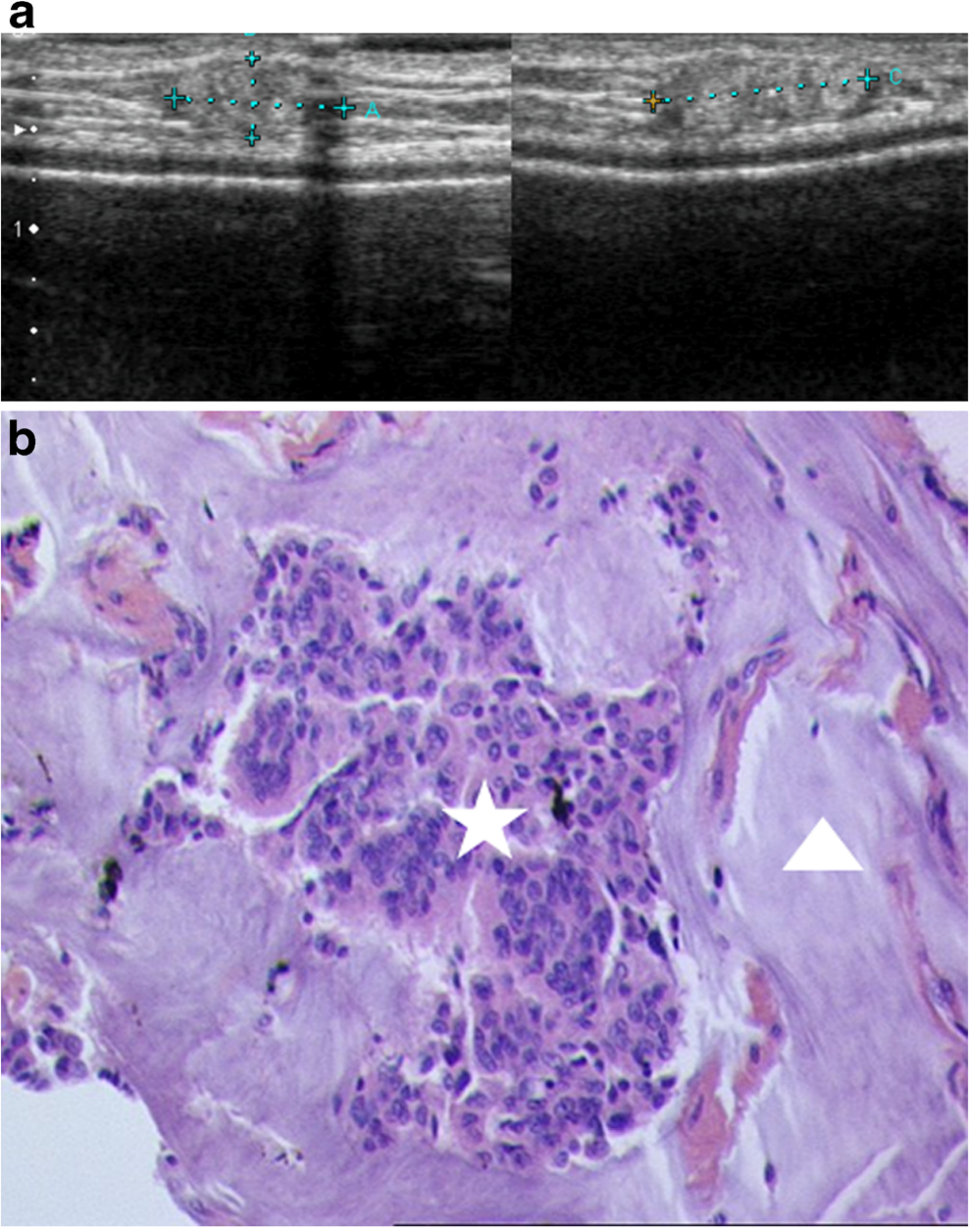
Mucinous carcinoma. Patient with a local subcutaneous recurrence in the upper inner quadrant, 10 years after mastectomy with secondary prosthetic reconstruction. **a** Ultrasound: hyperechoic mass of the subcutaneous tissue, with slightly microlobular margins. **b** Histological examination of an ultrasound-guided microbiopsy (HES ×20): clumps of tumour cells (*asterisk*) surrounded by zones of mucin (*arrowhead*)

Mucinous carcinoma (or colloid carcinoma) is an uncommon entity, accounting for between 1 and 7% of all breast cancers [18], with an increasing prevalence with age. Mucinous carcinomas can be subdivided into two histological subtypes, pure or mixed, depending on the mucin content [19]. On mammography, mucinous carcinoma usually presents in the form of a well-circumscribed, lobular or microlobular oval mass. Ultrasound classically reveals a hypoechoic or isoechoic, rarely hyperechoic, microlobular mass (Fig. 15).

### Invasive lobular carcinoma (ILC)

**Fig. 16:**
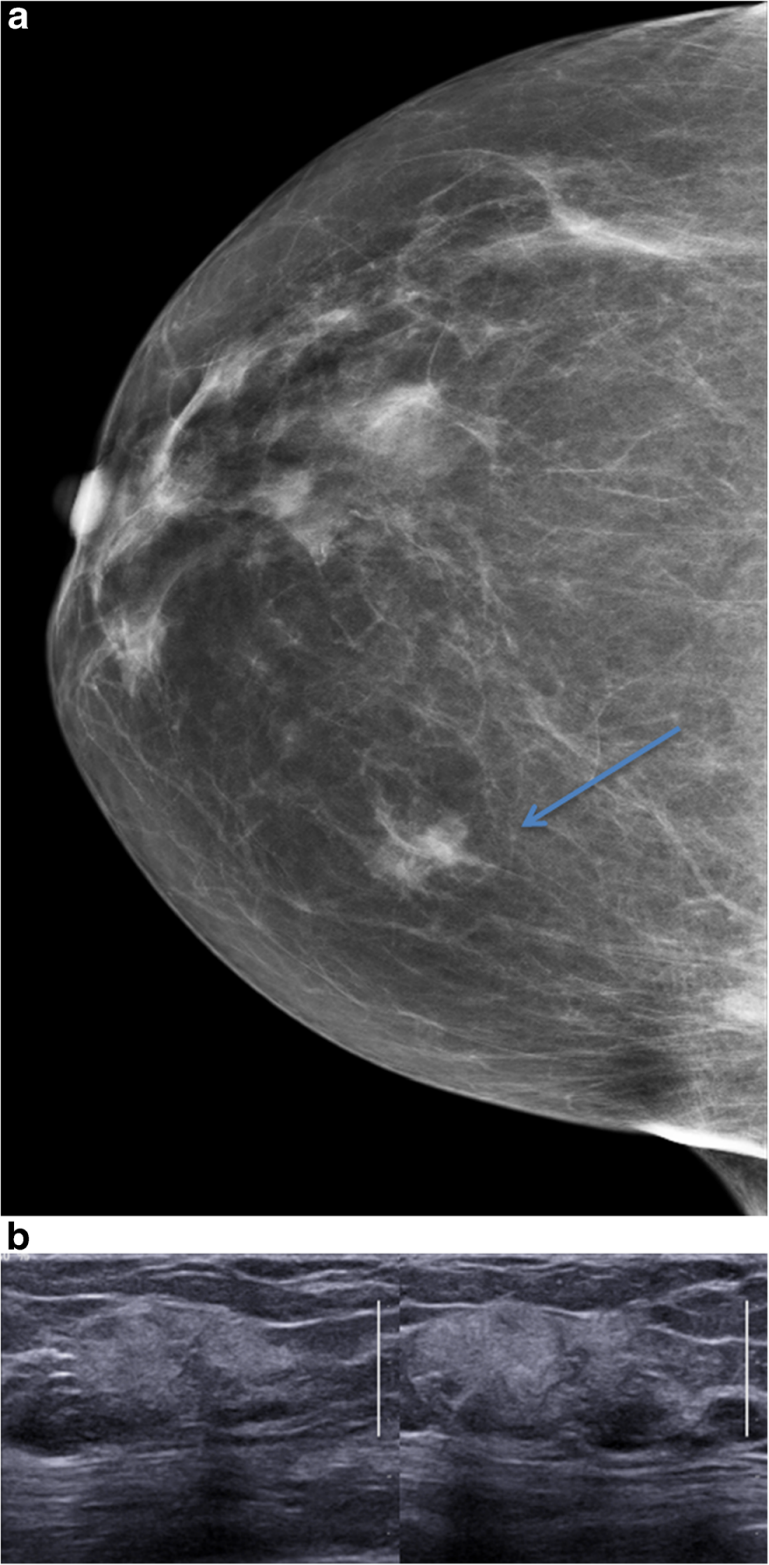
Invasive lobular carcinoma. **a** Mammography, AP view, showing a mass with irregular margins in the right upper inner quadrant (*arrows*). **b** Ultrasound showing an irregular, poorly circumscribed, vertically oriented, hyperechoic and partially attenuating lesion

ILC is the second most common form of breast carcinoma after invasive ductal carcinoma, characterised by tumour cells invading the breast parenchyma either separately or in chains. The most commonly observed mammographic appearance is that of a spiculated mass, but asymmetric density and architectural disorganisations are observed more frequently than in IDC (Fig. 16). As for IDC, ultrasound usually reveals a hypoechoic mass with spiculated margins and posterior attenuation. In contrast, ILC can present atypical characteristics such as the absence of a clearly defined mass or hyperechogenicity. Hyperechoic presentations are 10 times more frequent in ILC than in IDC [20].

### Angiosarcoma

Angiosarcoma is a rare, aggressive malignant tumour, accounting for less than 1% of all breast cancers. Two forms are distinguished: primary angiosarcoma, which is sporadic in young women, and secondary angiosarcoma in an irradiated breast, occurring an average of 6 years after radiotherapy.

**Fig. 17:**
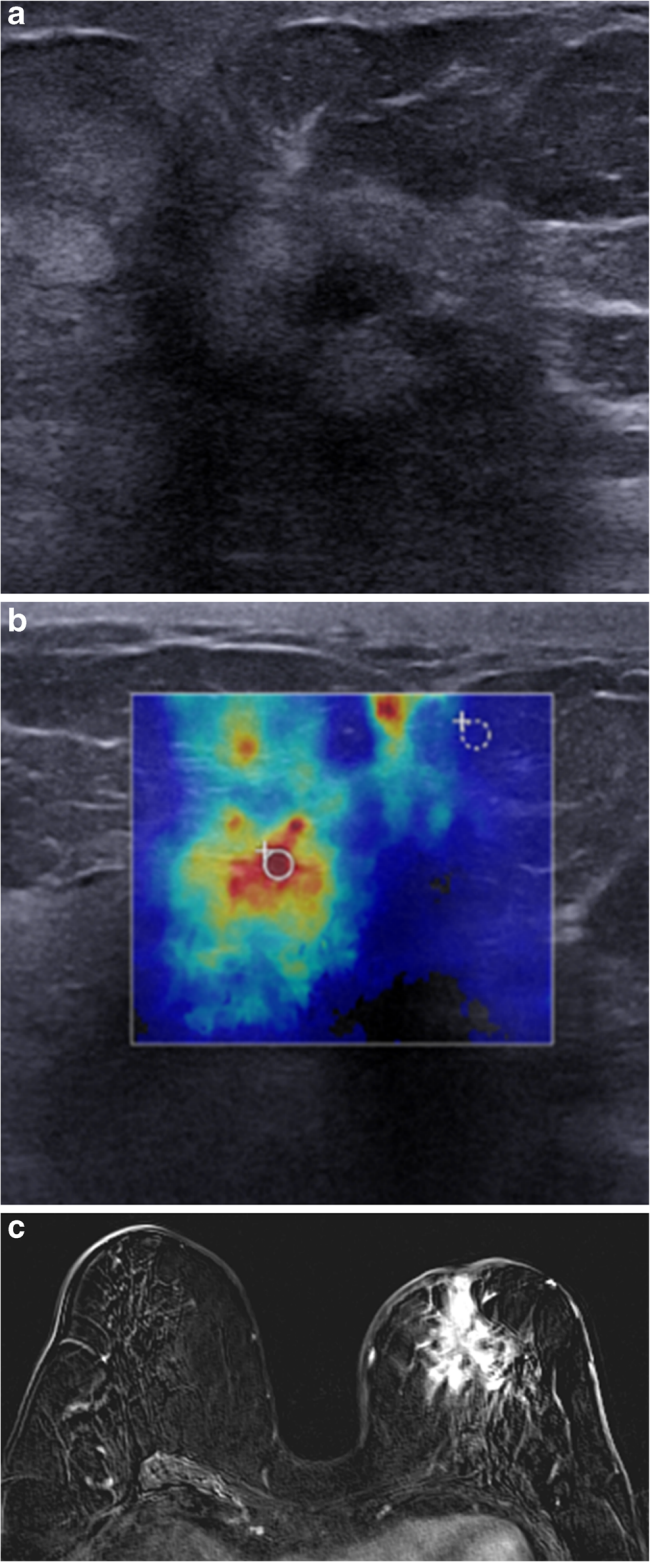
Angiosarcoma. A 76-year-old woman, treated conservatively for left breast cancer in 1993. Palpation of an upper quadrant breast swelling. **a** Ultrasound: heterogeneous, hyperechoic mass, with a hypoechoic centre. **b** Elastography, zones of hardness (*red*) in the periphery of the mass. **c** MRI, axial gadolinium-enhanced T1 subtraction sequence: spiculated mass with heterogeneous intense contrast enhancement. Histological examination revealed a high-grade moderately to poorly differentiated angiosarcoma

Angiosarcoma arises in the breast parenchyma (in contrast to haemangioma). It is a large tumour, often exceeding 3 cm at diagnosis. Mammography reveals a poorly demarcated, non-calcified mass, or focal asymmetric density. On ultrasound, angiosarcoma tends to present as isolated or multiple heterogeneous, hypoechoic, hypervascular masses, with irregular margins. Forty-four percent of angiosarcomas appear as hyperechoic or mixed hypoechoic and hyperechoic tumours [21] (Fig. 17).

### Metastases

**Fig. 18:**
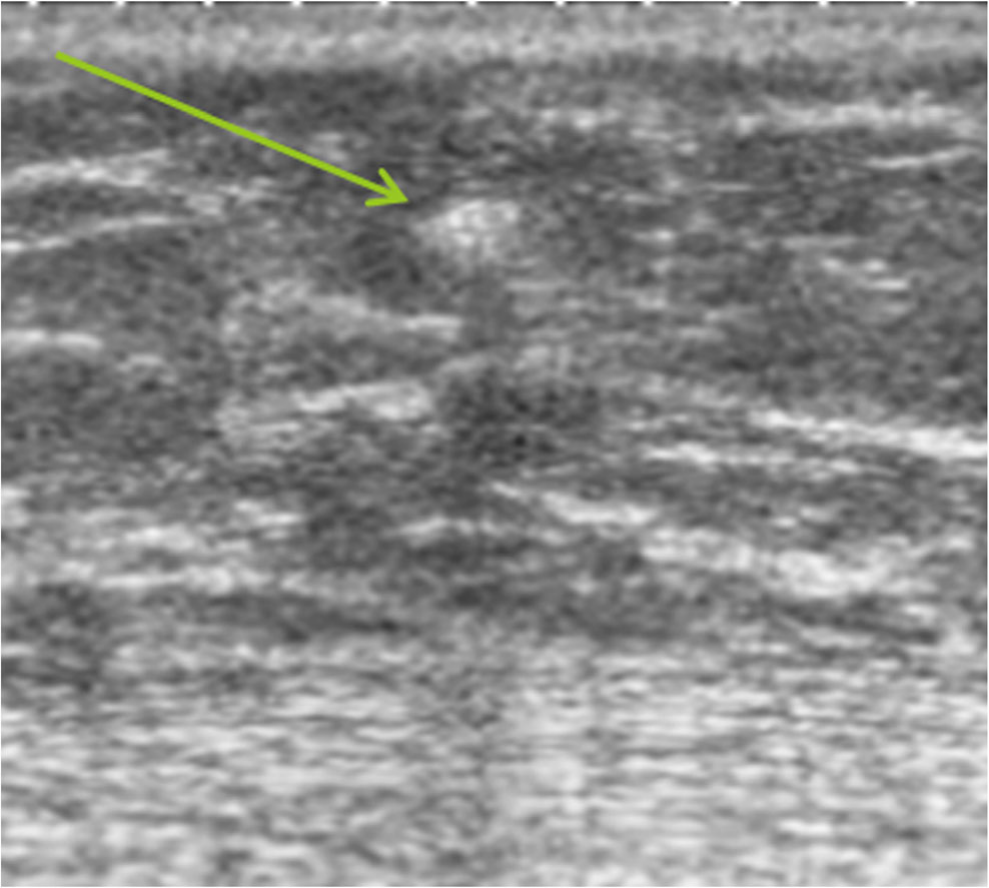
Metastasis. Ultrasound showing a small hyperechoic, attenuating mass (*arrow*) in a patient followed for malignant melanoma. Cytological examination after ultrasound-guided fine-needle aspiration: very numerous spindle-shaped, pigmented, round malignant cells, corresponding to a metastasis from known malignant melanoma in this patient

Metastases to the breast are rare, with the most common primary tumours being lung cancer, malignant melanoma, ovarian cancer, thyroid cancer, lymphoma and rhabdomyosarcoma. Mammography shows usually well-circumscribed, non-spiculated masses. Ultrasound usually shows more or less well-circumscribed hypoechoic or sometimes hyperechoic masses (Fig. 18) with attenuation or acoustic shadow. Skin metastases from malignant melanoma are typically hypervascular on Doppler studies.

### Lymphoma

Primary lymphoma of the breast is very rare, accounting for less than 0.5% of all breast cancers and less than 1% of non-Hodgkin’s malignant lymphomas, predominantly corresponding to diffuse B-cell lymphoma. Secondary lymphoma, more frequent, is associated with extramammary involvement at diagnosis.

**Fig. 19:**
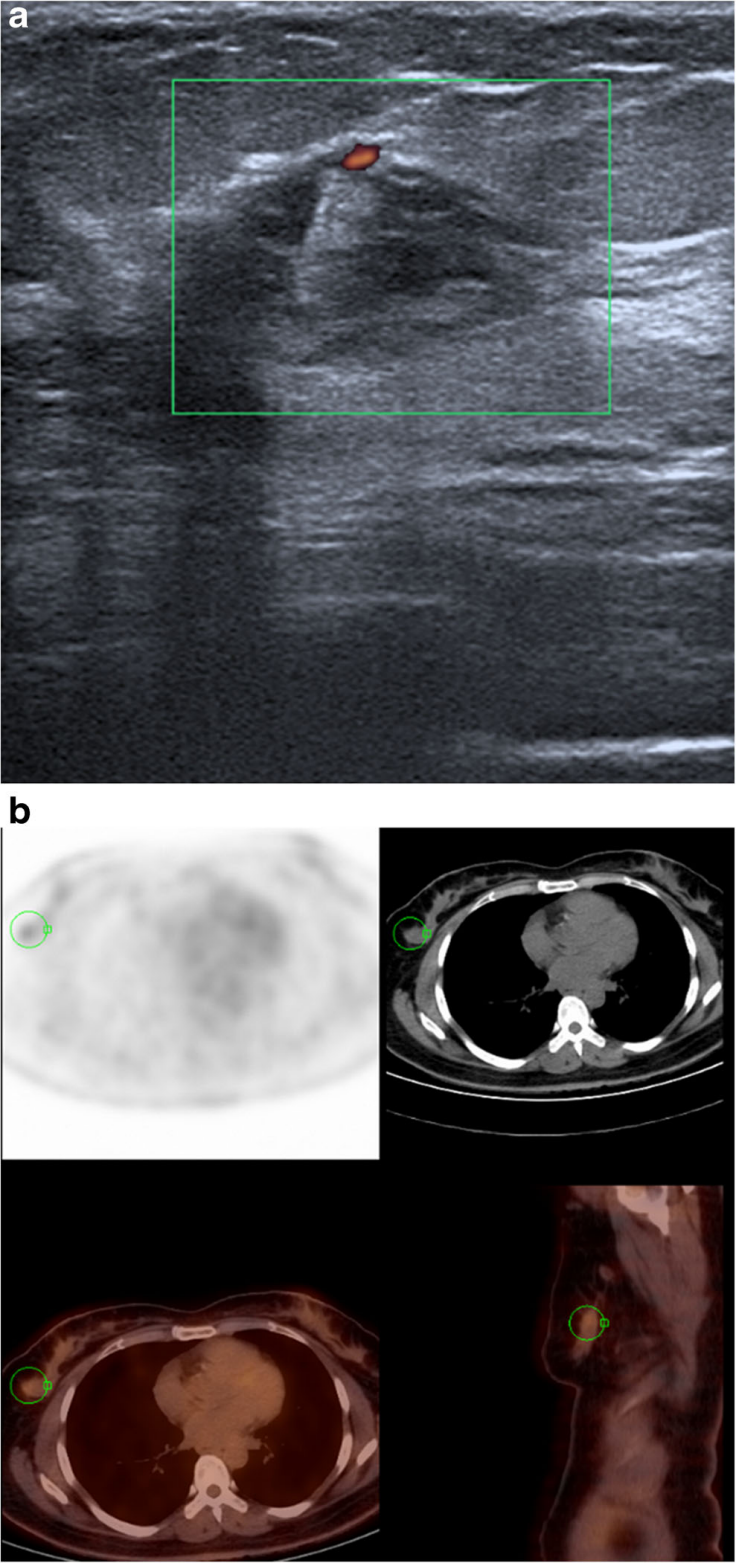
Lymphoma. **a** Ultrasound of the right breast showing a poorly demarcated, mixed echogenicity mass, with blood flow on Doppler ultrasound. **b** PET-CT scan in this patient shows a hypermetabolic lesion in the right breast. Microbiopsy revealed diffuse large B-cell lymphoma

Lymphoma is generally observed in old patients, revealed by a painful, palpable mass with local inflammation, sometimes associated with palpable axillary lymph nodes. Mammography reveals an irregular or lobular non-calcified mass. Multiple masses can also be responsible for asymmetric density. Ultrasound usually reveals a more or less well-circumscribed, hypervascular, hypoechoic mass or sometimes a mixed echogenicity mass, with a hyperechoic periphery (Fig. 19). Hyperechogenicity and mixed echogenicity can be seen in about 23% of breast lymphomas. According to some authors, the presence of hypervascularisation in a hyperechoic mass on Doppler ultrasound justifies biopsy [7, 22].

## Conclusions

The very great majority of hyperechoic masses of the breast are benign, and the diagnosis is often guided by the clinical setting. Most malignant lesions are hypoechoic compared to fat, especially when using the harmonic mode. However, malignant lesions can sometimes present in the form of hyperechoic or mixed echogenicity images, in which case other signs suggestive of malignancy are generally present: a more vertical axis, irregular shape, spiculated margins, posterior attenuation or hypervascularisation. Second-look post-MRI ultrasound may visualise hyperechoic malignant lesions that would not have been identified at first sight.
